# Response to the Letter: The NT‐proANP puzzle ‐ a small piece that makes the big picture

**DOI:** 10.1002/clc.23498

**Published:** 2020-11-14

**Authors:** Timm Seewöster, Jelena Kornej

**Affiliations:** ^1^ Department of Electrophysiology Heart Center Leipzig at University of Leipzig Leipzig Germany; ^2^ Boston University, School of Medicine—Cardiovascular Medicine Boston University Boston Massachusetts USA

To the Editor,

The challenges related to prediction of low voltage areas (LVAs) remain important for clinicians and scientists. Therefore, we appreciate the interest demonstrated by Floria and colleagues regarding our paper.^1^

Based on our previous investigation, prediction of electro‐anatomical substrate before catheter ablation seems to be more important for individualized AF management than arrhythmia recurrences, which partly depends on ablators' experience and follow‐up strategies.^2^ However, we agree that late gadolinium enhancement (LGE) and presence of LVAs do not necessarily overlap. Indeed, there are some technical issues and anatomical challenges for the electro‐anatomical mapping. Thus, LGE assessment could be challenging by differences in image acquisition and postprocessing protocols as well.^3^ Despite being a gold standard for imaging modality in cardiology, cardiac magnetic resonance (CMR)‐derived LA parameters are comparable with echocardiographic measurements.^4^ Also, randomized studies clearly favoring LGE as an ablation target have not been published yet, and the results still remain inconsistent.

AF causes multifactorial pathophysiological changes mirrored by alteration in left atrial (LA) anatomy and function, impaired atrial conduction, and disturbances in blood biomarkers' profiles just to name few. Importance of LA volume^5^ and LA function^6^ for LVAs prediction had been already shown. Floria et al hypothesize that NT‐proANP secretion might decrease due to less secreting myocardium caused by increase of LA volume and LVAs presence. However, our previous findings clearly contradict this assumption, as we were able to show almost a linear increase in the NT‐proANP levels accordingly to four AF progression phenotypes.^7^ Similar association was found not only for echocardiographic LA volume,^7^ but also for CMR‐derived LA size.^5^

In summary, further research elucidating non‐invasive prediction of LVAs presence is clearly needed. Advanced imaging modalities, high‐fidelity ECG, and/or specific blood biomarkers should be implemented into individualized AF management and disease prevention.

## Data Availability

Data sharing is not applicable to this article as no new data were created or analyzed in this study.

## References

[1] Seewöster T , Büttner P , Zeynalova S , Hindricks G , Kornej J . Are the atrial natriuretic peptides a missing link predicting low‐voltage areas in atrial fibrillation? Introducing the novel biomarker‐based atrial fibrillation substrate prediction (ANP) score. Clin Cardiol. 2020;43(7):762‐768. 10.1002/clc.23378. 32462768 PMC7368300

[2] Kornej J , Schumacher K , Zeynalova S , et al. Time‐dependent prediction of arrhythmia recurrences during long‐term follow‐up in patients undergoing catheter ablation of atrial fibrillation: The Leipzig Heart Center AF Ablation registry. Sci Rep. 2019;9(1):7112 10.1038/s41598-019-43644-2. 31068651 PMC6506496

[3] Benito EM , Carlosena‐Remirez A , Guasch E , et al. Left atrial fibrosis quantification by late gadolinium‐enhanced magnetic resonance: a new method to standardize the thresholds for reproducibility. Europace. 2017;19(8):1272‐1279. 10.1093/europace/euw219. 27940935

[4] Seewöster T , Kosich F , Sommer P , et al. Prediction of low voltage areas using modified APPLE score. Europace. 2020 10.1093/europace/euaa311 In press. 33279992

[5] Seewöster T , Büttner P , Nedios S , et al. Association between cardiovascular magnetic resonance‐derived left atrial dimensions, electroanatomical substrate and NT‐proANP levels in atrial fibrillation. J Am Heart Assoc. 2018;7(19):e009427 10.1161/JAHA.118.009427. 30371296 PMC6404891

[6] Seewöster T , Spampinato RA , Sommer P , et al. Left atrial size and total atrial emptying fraction in atrial fibrillation progression. Heart Rhythm. 2019;16(11):1605‐1610. 10.1016/j.hrthm.2019.06.014. 31228634

[7] Büttner P , Schumacher K , Dinov B , et al. Role of NT‐proANP and NT‐proBNP in patients with atrial fibrillation: association with atrial fibrillation progression phenotypes. Heart Rhythm. 2018;15(8):1132‐1137. 10.1016/j.hrthm.2018.03.021. 29604419

